# Energy Transfer in Ce_0.85_Tb_0.15_F_3_ Nanoparticles-CTAB Shell-Chlorin e_6_ System

**DOI:** 10.1186/s11671-017-2077-x

**Published:** 2017-04-24

**Authors:** Mykhaylo Yu. Losytskyy, Liliia V. Kuzmenko, Oleksandr B. Shcherbakov, Nikolai F. Gamaleia, Andrii I. Marynin, Valeriy M. Yashchuk

**Affiliations:** 1 Taras Shevchenko National University of Kyiv, Volodymyrska Str., 64/13, Kyiv, 01601 Ukraine; 2 Zabolotny Institute of Microbiology and Virology, NAS of Ukraine, 154 Zabolotnogo Str., Kyiv, 03680 Ukraine; 3 R.E. Kavetsky Institute for Experimental Pathology, Oncology and Radiobiology, NAS of Ukraine, 45 Vasylkivska Str., Kyiv, 03022 Ukraine; 4 National University of Food Technologies, Volodymyrska Str. 68, Kyiv, 01601 Ukraine

**Keywords:** Ce_0.85_Tb_0.15_F_3_ nanoparticles, Chlorin e_6_, Electronic excitation energy transfer, Photodynamic therapy

## Abstract

Formation and electronic excitation energy transfer process in the nanosystem consisting of Ce_0.85_Tb_0.15_F_3_ nanoparticles, cetrimonium bromide (CTAB) surfactant, and chlorin e_6_ photosensitizer were studied. It was shown that chlorin e_6_ molecules bind to Ce_0.85_Tb_0.15_F_3_ NP in the presence of CTAB forming thus Ce_0.85_Tb_0.15_F_3_ NP-CTAB-chlorin e_6_ nanosystem. We consider that binding occurs via chlorin e_6_ embedding in the shell of CTAB molecules, formed around NP. In the Ce_0.85_Tb_0.15_F_3_ NP-CTAB-chlorin e_6_ nanosystem, electronic excitation energy transfer from Ce^3+^ to chlorin e_6_ takes place both directly (with the 0.33 efficiency for 2 μM chlorin e_6_) and via Tb^3+^.

## Background

Photodynamic therapy (PDT) is the method for the treatment of cancer, where the photosensitizer excitation by the light leads to the generation of singlet oxygen that is toxic for the tumor tissue [1]. But despite of several advantages, the drawback of this method is the very small depth of the light penetration into the tissue [2]. Thus, the idea of X-ray-inducible PDT based on X-ray-excited sensitizers composed of scintillating and photosensitizing parts with the electronic excitation energy transfer (EEET) from the first to the last one seems attractive [3]. Recently, a number of nanosystems based on this concept were described with various materials used as scintillators, different photosensitizer molecules, and several ways of binding them into a system [2, 4–9]; X-rays induced singlet oxygen generation [2, 6, 7, 9], cell destruction [4, 5, 7], and tumor destruction in mice [7] were demonstrated.

One of the options to choose scintillator for the above-described nanosystems is using lanthanide fluoride nanoparticles (NPs) [5, 9, 10]. Since f-f transitions of the majority of lanthanides are strongly forbidden, while in the case of Ce^3+^ its d-f transitions are allowed, generally, the lanthanide ions except Ce^3+^ cannot be efficiently excited by light in the UV-visible spectral region [11]. Thus, for the study of photophysics properties, CeF_3_-based [8, 12, 13] or Ce^3+^-doped [8, 10, 11] nanoparticles are used; excitation of Ce^3+^ results either in its own emission or in that of another lanthanide ion dopant (e.g., Tb^3+^) due to EEET [12, 13]. Excitation energy transfer from lanthanide nanoparticles to electrostatically bound [8, 9] or covalently attached [8, 10] photosensitizers was demonstrated.

Chlorin e_6_ is a known compound with photosensitizing properties used in PDT of cancer [14, 15]; its combination with scintillating lanthanide fluoride NP could be promising for the X-ray-inducible PDT. Nanosystems containing conjugates of La_0.9_Ce_0.1_F_3_ to chlorin e_6_ were studied in [10], though Tb-doped NPs were not investigated with chlorin e_6_ because of poor spectral overlap [10]. At the same time, EEET from Tb-doped NP to protoporphyrin IX was demonstrated in [16]. Thus, the possible role of Tb^3+^ doping agent in EEET pathways taking place in nanosystems containing lanthanide fluoride NP and chlorin e_6_ photosensitizer should be studied. X-ray-induced scintillation emission spectra of CeF_3_ NP (both undoped and Tb^3+^-doped) were similar to their photoluminescence spectra excited in ultraviolet spectral region [9, 16–18]. EEET processes in Tb^3+^-doped cerium fluoride NP are also expected to be similar for the cases of X-rays and ultraviolet light excitation. Therefore, studies of energy transfer between the sensitizer and Tb^3+^-doped CeF_3_ NP can be carried out using UV excitation as a model. Here, we study formation and EEET process in the nanosystem consisting of Ce_0.85_Tb_0.15_F_3_ nanoparticles, cetrimonium bromide (CTAB) surfactant, and chlorin e_6_ photosensitizer.

## Methods

### Materials

Hydrofluoric acid, isopropyl alcohol, cerium(III) chloride heptahydrate, and terbium(III) chloride heptahydrate were acquired at Sigma-Aldrich Co. and used without further purification. Chlorin e_6_ (Frontier Scientific Inc.) was kindly provided by T.Y. Ohulchanskyy (Institute for Lasers, Photonics and Biophotonics at the State University of New York at Buffalo). Fifty millimolar TRIS-HCl buffer (pH 7.2) was used as solvent.

### Synthesis and Characterization of Nanoparticles

Ce_0.85_Tb_0.15_F_3_ NPs (0.07 M water solution) were synthesized as described in [19]. Briefly, a mixture of 1.58 g of cerium(III) chloride heptahydrate (4.25 mmol, i.e., 85%) and 0.293 g of terbium(III) chloride heptahydrate (0.75 mmol, i.e., 15%) was dissolved in 15 mL of water and added to 150 mL of isopropyl alcohol. Hydrofluoric acid (20 mmol), dissolved in 50 mL of isopropyl alcohol, was added drop-wise to a cerium and terbium salt solution under vigorous stirring. The resulting white sediment was filtered and washed carefully by pure isopropyl alcohol several times. Then, the suspension was slightly dried to form a paste-like substance and dispersed in 110 ml of distilled water using an ultrasonic bath. The resulting transparent colloid solution was boiled for 5 min to remove residual alcohol.

**Fig. 1:**
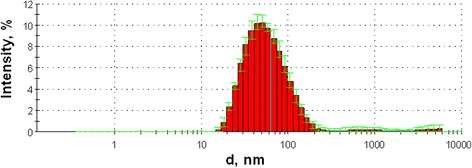
Distribution of DLS intensity for Ce_0.85_Tb_0.15_F_3_ nanoparticles, averaged for 5 measurements

Particle size distribution was studied by the dynamic light scattering (DLS) technique using ZetasizerNano ZS (Malvern Instruments) apparatus (Fig. 1). For the obtained Ce_0.85_Tb_0.15_F_3_ NP, intensity distribution of the hydrodynamic diameter gave main maximum at 62 ± 36 nm (about 97% of intensity) with negligibly small addition of larger fractions. The Z potential of the synthesized NPs was determined as +41 ± 14 mV.

**Fig. 2:**
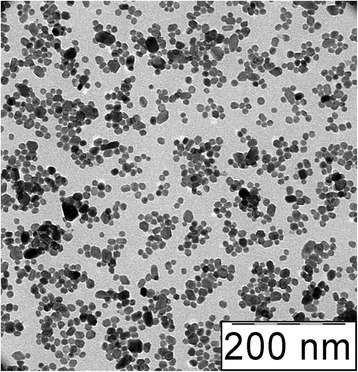
TEM image of Ce_0.85_Tb_0.15_F_3_ nanoparticles

A representative transmission electron microscopy (TEM) image of Ce_0.85_Tb_0.15_F_3_ nanoparticles obtained in the above-described reaction is provided in Fig. 2. TEM was performed using a Leo 912 AB Omega electron microscope operating at 100 kV. Before the analysis, sols were brought onto the copper grids using micropipette without any specific pretreatment and dried in ambient air. Comparison of Figs. 1 and 2 shows that the size of the nanoparticles obtained by TEM is smaller than the hydrodynamic diameter at the maximum of DLS intensity distribution. We believe this is connected with the peculiar property of the DLS method that the DLS intensity by a particle is proportional to the sixth power of its radius; thus, larger particle makes higher contribution to the DLS intensity as compared to smaller one. Recalculation of the obtained intensity distribution to nanoparticle number distribution according to the mentioned sixth power relation resulted in a maximum at 24 ± 7 nm that is in agreement with the TEM results.

### Preparation of Samples

Concentrated solution of chlorin e_6_ (10 mM) was prepared in DMF. To prepare the solution of the studied nanosystems, an aliquot (20 μL per 1 mL) of 0.07 M water solution of﻿ Ce_0.85_Tb_0.15_F_3_ nanoparticles was added to the CTAB solution (0.05 mg/mL CTAB concentration was found to be optimal) in 50 mM TRIS-HCl buffer (pH 7.2). An aliquot of chlorin e_6_ concentrated solution was then added; in order to minimize reabsorption, 2 μM concentration of chlorin e_6_ was used; at this concentration, chlorin e_6_ has negligible absorption at the maximum wavelength of Ce^3+^ emission, while at the wavelength of the Soret band maximum (near 400 nm; optical density about 0.3 for the used concentration of chlorin e_6_), Ce^3+^ emission is already weak. Besides, 5 and 10 μM concentrations of chlorin e_6_ were additionally used in Tb^3+^ luminescence decay measurements. Solution of chlorin e_6_ (2 μM) in the presence of concentrated micelles-forming CTAB (1 mg/mL) was used for the comparison.

### Spectral Measurements

Absorption spectra were measured using Specord M40 spectrophotometer (Carl Zeiss, Germany). Luminescence excitation, emission, and anisotropy spectra as well as the curves of luminescence decay in millisecond timescale were registered with the help of the Cary Eclipse fluorescent spectrophotometer (Varian, Australia). Absorption and fluorescence measurements were performed in 1 cm × 1 cm quartz cell at room temperature. Quantitative estimation of the efficiency of Ce^3+^ to chlorin e_6_ EEET (E_Ce−Ce6_) was performed as described in [20] by comparison of chlorin e_6_ fluorescence intensities upon excitation of Ce^3+^ (${I}_{{\mathrm{emCe}}_6}^{{\mathrm{exCe}}^{3+}}$, contribution to this intensity of the own excitation of chlorin e_6_ at this wavelength was subtracted) and chlorin e_6_ itself (${I}_{{\mathrm{emCe}}_6}^{{\mathrm{exCe}}_6}$; optical densities of Ce^3+^ and chlorin e_6_ at the used excitation wavelengths were equal) according to:

1 $$ {E}_{Ce- Ce6}=\frac{I_{{\mathrm{emCe}}_6}^{{\mathrm{exCe}}^{3+}}}{I_{{\mathrm{emCe}}_6}^{{\mathrm{exCe}}_6}} $$

The value of *E*_Ce−Ce6_ could be also estimated by comparison of the integral intensities of Ce^3+^ emission in the presence (Int_NPCe6_) and in the absence (Int_NP_) of chlorin e_6_, given that the reabsorption could be neglected, as:

2 $$ {E}_{\mathrm{Ce}-\mathrm{Ce}6}=1-\frac{{\mathrm{Int}}_{\mathrm{NP}\;{\mathrm{Ce}}_6}}{{\mathrm{Int}}_{\mathrm{NP}}} $$

When performing estimation of the efficiency of Ce^3+^-to-chlorin e_6_ EEET by (1) and (2), spectral sensitivity of the fluorescent spectrophotometer on the excitation and emission wavelength was taken into account.

Decay curves of Tb^3+^ luminescence were fitted by three exponents; the relative intensities *B*_1_, *B*_2_, and *B*_3_ are calculated as *B*_*i*_ = *A*_*i*_ × *τ*_*i*_/Σ *A*_i_ × τ_i_ (*i* = 1, 2, 3; *τ*_1_, *τ*_2_, and *τ*_3_ are the decay times; *A*_1_, *A*_2_, and *A*_3_ are amplitudes of corresponding exponents). Quantitative estimation of the efficiency of Tb^3+^-to-chlorin e_6_ EEET for each of the three decay components (*E*_Tb−Ce6_(*τ*_*i*_)) was performed by comparison of the decay times of corresponding components of Tb^3+^ luminescence at 543 nm in the absence of chlorin e_6_ (*τ*_*i*_^Tb^) and in its presence (*τ*_*i*_^TbCe6^) as:

3 $$ {E}_{\mathrm{Tb}-\mathrm{Ce}6}\left({\tau}_i\right)=1-\frac{\tau_i^{{\mathrm{Tb}\mathrm{Ce}}_6}}{\tau_i^{\mathrm{Tb}}} $$

Amplitudes *A*_1_, *A*_2_, and *A*_3_ are affected (to different extent for different components) by addition of chlorin e_6_ due to decreased EEET from Ce^3+^ to Tb^3+^. Thus, the expression (3) cannot be applied for calculation of total Tb^3+^-to-chlorin e_6_ EEET efficiency using average decay time values.

Three-exponential fit of the Tb^3+^ decay curves was also used to estimate real decrease of Tb^3+^ luminescence intensity upon addition of chlorin e_6_. Cary Eclipse fluorescent spectrophotometer uses pulsed xenon lamp (80 Hz; 2 μs pulse width at half peak height) for luminescence excitation, setting the intensity measured before each pulse (thus, in about 12.5 ms after previous pulse) as zero for the background correction. Hence, the intensity of Tb^3+^ luminescence that has decay times in ms range is artificially reduced; but addition of chlorin e_6_ leads to the decay time decrease and thus less significant artificial reduction of the intensity. So, the Tb^3+^ luminescence quenching apparent from the spectra is less strong than the real one. Thus, we estimated the real intensities of Tb^3+^ luminescence (and thus its real decrease upon chlorin e_6_ addition) using the results of the three-exponential fit of the luminescence decay curve as *A*_1_ × *τ*_1_ + *A*_2_ × *τ*_2_ + *A*_3_ × *τ*_3_.

## Results and Discussion

### EEET in NP-CTAB-Chlorin e_6_ Nanosystem

First of all, it should be mentioned that it is not possible to prepare the nanosystem with EEET consisting of the synthesized Ce_0.85_Tb_0.15_F_3_ NP and the monomer form of chlorine e_6_ based only on electrostatic interactions without any linking group or some binding substance. While in the distilled water, chlorin e_6_ molecules form aggregates on Ce_0.85_Tb_0.15_F_3_ NP that leads to complete quenching of the chlorin e_6_ fluorescence; in 50 mM TRIS-HCl buffer (pH 7.2), no manifestation of interaction was observed in chlorin e_6_ absorption and emission spectra. Such dependence on medium should be connected with the change in chlorin e_6_ molecule taking place at pH value about 6.1 [14].

**Fig. 3:**
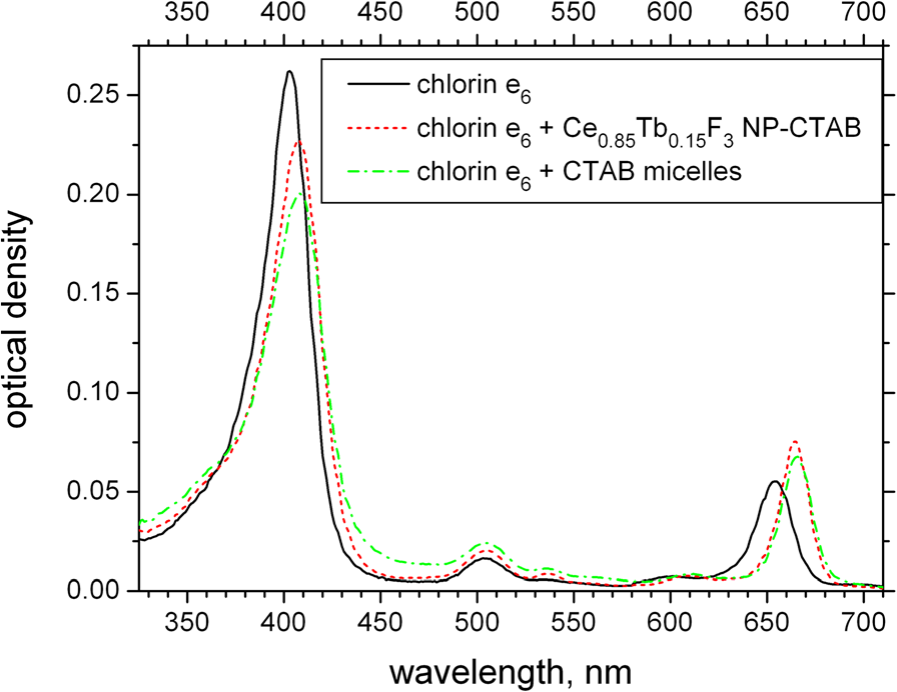
Absorption spectra of 2 μM chlorin e_6_ free in solution (*black solid line*) and in the presence of CTAB micelles (1 mg/mL CTAB; *green dash-dotted line*) and Ce_0.85_Tb_0.15_F_3_ NP-CTAB-chlorin e_6_ nanosystem (2 μM chlorin e_6_; 0.05 mg/mL CTAB; *red dashed line*)

**Fig. 4:**
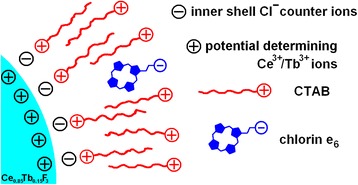
Schematic representation of the supposed arrangement of the components in the NP-CTAB-chlorin e_6_ nanosystem. In the symbol representing chlorin e_6_ molecule, the *minus* placed in the circle stands for the net negative charge of the molecule that could be due to some or all of its three carboxylic groups. Bromide counter ions of the CTAB molecules are not shown for simplicity

In order to form model nanosystem containing Ce_0.85_Tb_0.15_F_3_ NP and chlorin e_6_, surfactant CTAB was used that was reported as a stabilizer for lanthanide fluoride NPs in [11]. Thus, to the solution of NPs in the presence of 0.05 mg/mL CTAB, 2 μM of chlorin e_6_ was added. Absorption spectrum of the obtained solution in comparison with these of chlorin e_6_ free and in the presence of CTAB micelles (i.e., CTAB of the concentration 1 mg/mL) is presented in Fig. 3. Absorption spectra of chlorin e_6_ in the presence of CTAB micelles and CTAB-NP nanosystems are similar but significantly different from chlorin e_6_ spectrum in buffer. Thus, we could suppose that in both cases, chlorin e_6_ molecules are built in a shell formed by CTAB molecules; supposed arrangement of the components in the NP-CTAB-chlorin e_6_ nanosystem is schematically presented in Fig. 4.

**Fig. 5:**
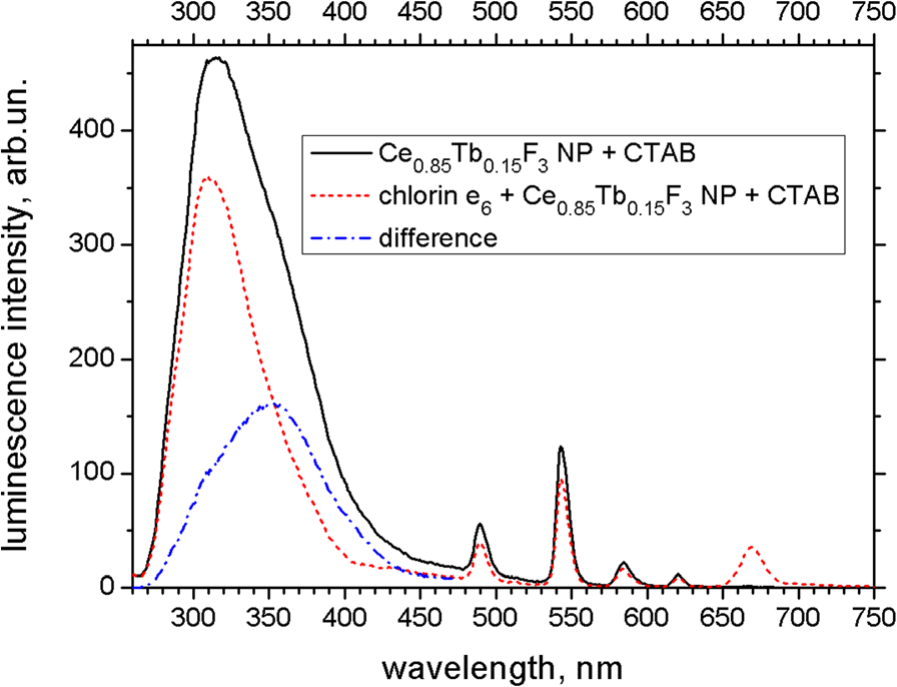
Fluorescence emission spectra of Ce_0.85_Tb_0.15_F_3_ NP in the presence of CTAB (0.05 mg/mL CTAB; *black solid line*), Ce_0.85_Tb_0.15_F_3_ NP-CTAB-chlorin e_6_ nanosystem (2 μM chlorin e_6_; 0.05 mg/mL CTAB; *red dashed line*), and difference of Ce^3+^ bands in these spectra (*blue dash-dotted line*). Excitation wavelength 250 nm

**Fig. 6:**
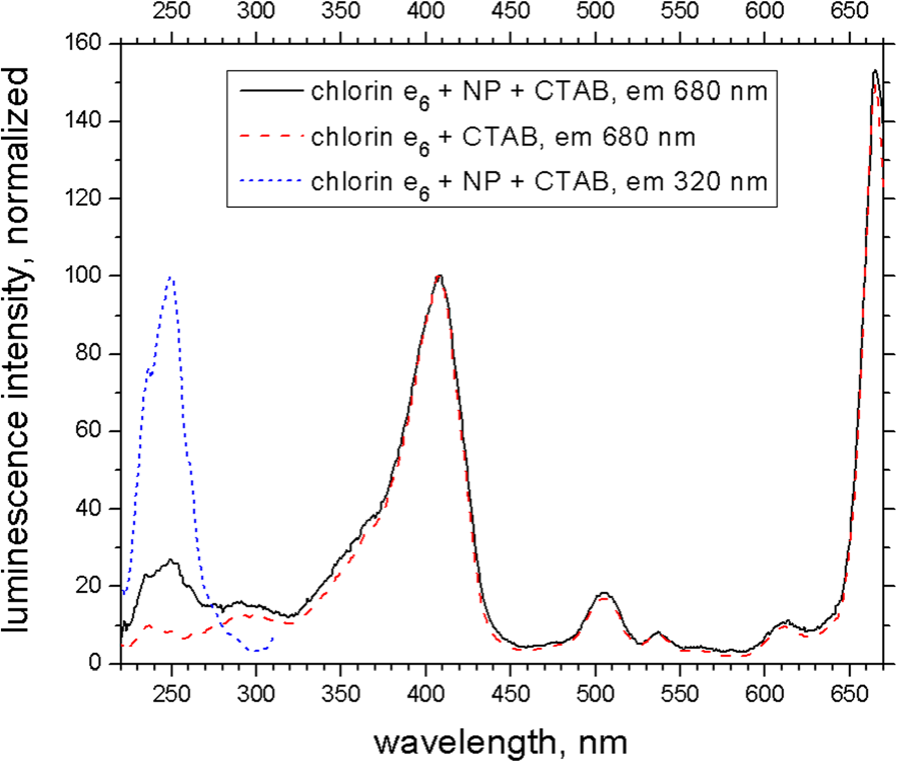
Normalized fluorescence excitation spectra of Ce_0.85_Tb_0.15_F_3_ NP-CTAB-chlorin e_6_ nanosystem (2 μM chlorin e_6_; 0.05 mg/mL CTAB; *black solid line* (emission of chlorin e_6_ at 680 nm) and *blue short dashed line* (emission of Ce^3+^ at 320 nm)) and chlorin e_6_ in the presence of CTAB micelles (1 mg/mL CTAB; emission at 680 nm; *red dashed line*)

Fluorescence emission spectra of Ce_0.85_Tb_0.15_F_3_ NP in the presence of CTAB (Fig. 5) demonstrate broad band corresponding to Ce^3+^ emission (320 nm) and narrow bands of Tb^3+^ ions (490, 543, 584, and 621 nm) as described in the literature [13]. Addition of chlorin e_6_ (Fig. 5) results in decrease of the intensity of Ce_0.85_Tb_0.15_F_3_ NP emission bands as well as in appearance of the band corresponding to chlorin e_6_ fluorescence (670 nm). This could be explained as EEET of Ce_0.85_Tb_0.15_F_3_ NP excitations to the chlorin e_6_ molecules bound to the CTAB shell of Ce_0.85_Tb_0.15_F_3_ NP. This conclusion could be also supported by fluorescence excitation measurements (Fig. 6). In the normalized fluorescence excitation spectra of chlorin e_6_ (emission at 680 nm) besides its own Soret and Q-bands, we observe the band at 250 nm, which coincides with the one in the excitation spectrum of Ce_0.85_Tb_0.15_F_3_ NP (emission at 320 nm); this is consistent with EEET from Ce^3+^ ions to chlorin e_6_. The increase in the fluorescence anisotropy of chlorin e_6_ in the presence of NP-CTAB (data not presented) is one more proof of the formation of NP-CTAB-chlorin e_6_ nanosystem.

It is seen from Fig. 5 that the addition of chlorin e_6_ leads to the narrowing and short-wavelength shift of the Ce^3+^ emission band. Difference of the unquenched and quenched Ce^3+^ bands gives the broad band with the maximum near 355 nm (Fig. 5) that most possibly corresponds to the emission of the perturbed Ce^3+^ states; these states were supposed to be the traps for the non-perturbed Ce^3+^ excitations transferring these excitations to either Tb^3+^ dopant or to the attached photosensitizer [8]. It should be added that the mentioned difference spectrum does not contain any significant component similar to that of Soret band of chlorin e_6_; thus, at these concentrations, the impact of reabsorption on the Ce^3+^ emission quenching by chlorin e_6_ could be considered as negligible. Based on Ce^3+^ emission spectra in the presence and in the absence of chlorin e_6_, efficiency of EEET could be estimated by comparing integral intensities of Ce^3+^ emission according to (2); the value of EEET efficiency equal to 0.33 was obtained for the 2 μM concentration of chlorin e_6_. Increasing the chlorin e_6_ concentration results in more significant EEET efficiency values, but these values also contain higher reabsorption contribution.

Efficiency of EEET from Ce^3+^ to chlorin e_6_ could be also estimated from the fluorescence excitation spectra by comparison of chlorin e_6_ fluorescence intensities upon excitation of Ce^3+^ (at 271 nm; contribution to this intensity of the own excitation of chlorin e_6_ at this wavelength was subtracted using the normalized excitation spectrum of chlorin e_6_ in CTAB micelles (Fig. 6)) and chlorin e_6_ itself (at 406 nm; optical densities of Ce^3+^ at 271 nm and chlorin e_6_ at 406 nm are equal) according to (1). Surprisingly, the value of EEET efficiency of about 0.06 was obtained that is much less than the value of 0.33 obtained according to (2). We could suppose that the EEET efficiency calculation based on (1) cannot be applied in our case. Perhaps Ce^3+^-to-chlorin e_6_ EEET brings chlorin e_6_ molecule to the vibronic levels with higher ability to further intersystem conversion as compared to the photoexcitation at 406 nm; this would cause decreased fluorescence quantum yield of chlorin e_6_ leading to lower values of  apparent EEET efficiency than calculated by (1).

It should be mentioned that the close proximity of Ce_0.85_Tb_0.15_F_3_ NP causes the strong decrease in the fluorescence intensity of chlorin e_6_; the same effect was noticed in [10]. The possible reason for this could be the heavy atom effect, i.e., more intensive transition of the excitations to the triplet state due to the close proximity of Ce and Tb atoms causing spin-orbit interactions in chlorin e_6_ molecule. One more possible explanation could be EEET between chlorin e_6_ molecules in the case where they are bound to NP-CTAB nanosystem at the mutual distances that are close enough for chlorin e_6_-chlorin e_6_ energy transfer.

### EEET Pathways in NP-CTAB-Chlorin e_6_ Nanosystem

It is interesting to study in more details the pathway of EEET from NP to chlorin e_6_. It is known that EEET from Ce^3+^ to Tb^3+^ ions takes place inside Ce_0.85_Tb_0.15_F_3_ nanoparticles [12, 13]. When adding chlorin e_6_ to the nanosystem, the following additional processes could occur besides the mentioned Ce^3+^-to-Tb^3+^ EEET: (i) EEET from Ce^3+^ perturbed states directly to chlorin e_6_ and (ii) EEET from excited Tb^3+^ ions to chlorin e_6_ molecules.

**Fig. 7:**
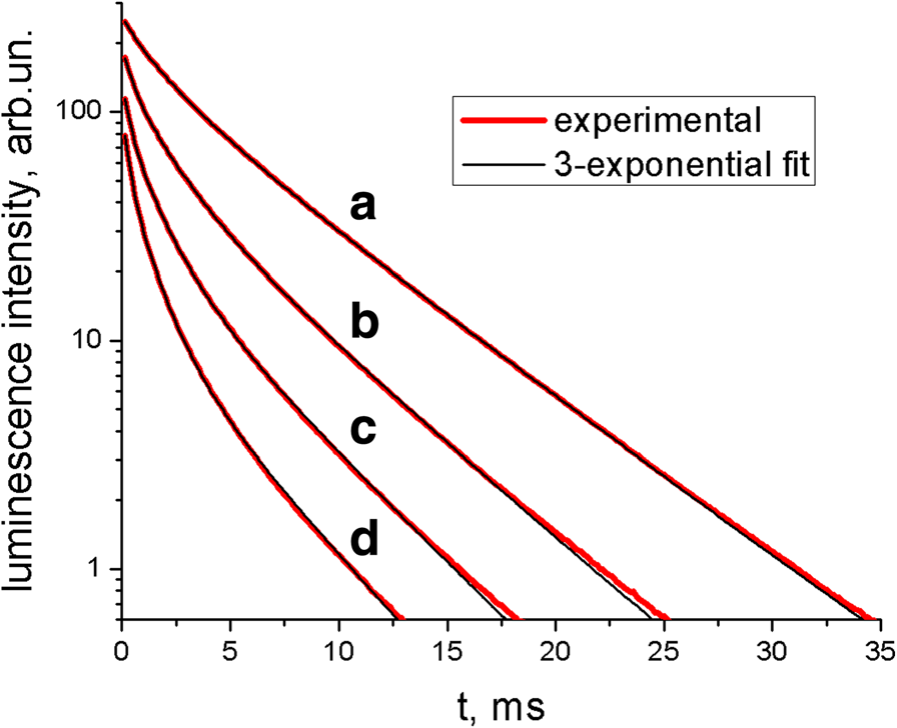
Luminescence decay curve (*broad red line*) and its three-exponential fit (*thin black line*) of Tb^3+^ band at 543 nm of Ce_0.85_Tb_0.15_F_3_ NP in the presence of 0.05 mg/mL CTAB (*a*) and Ce_0.85_Tb_0.15_F_3_ NP-CTAB-chlorin e_6_ nanosystem for 2 μM (*b*), 5 μM (*c*), and 10 μM (*d*) concentration of chlorin e_6_; CTAB concentration 0.05 mg/mL. Excitation wavelength 250 nm

First of all, since the excited state lifetime of Tb^3+^ ions is extremely long as compared to that of Ce^3+^ [8, 13], decrease in the Ce^3+^ emission intensity upon addition of chlorin e_6_ means the direct EEET from Ce^3+^ to chlorin e_6_ (with the 0.33 efficiency for 2 μM chlorin e_6_). Further, it is seen from Fig. 5 that together with the decrease in Ce^3+^ emission, this of Tb^3+^ diminishes as well. The apparent decrease in the Tb^3+^ emission band intensity upon addition of chlorin e_6_ is about 20–23%, but the total intensity values of the millisecond Tb^3+^ emission are biased by the spectrofluorometer (see "Spectral measurements" subsection﻿ in the “Methods” section); real intensity decrease was estimated using Tb^3+^ luminescence decay curves as 56% (543-nm band for 2 μM chlorin e_6_) exceeding that for Ce^3+^ (33% for 2 μM chlorin e_6_). Thus, the Tb^3+^ emission quenching could be due both to (i) Tb^3+^-to-chlorin e_6_ EEET and to (ii) decreased Ce^3+^-to-Tb^3+^ EEET (and thus reduced population of excited levels of Tb^3+^) caused by competition with the direct Ce^3+^-to-chlorin e_6_ EEET. The observed decrease in the Tb^3+^ emission decay time upon the addition of chlorin e_6_ (Fig. 7) points to the existing of EEET from excited Tb^3+^ ions to chlorin e_6_ molecules; such transfer was also reported for protoporphyrin IX in [16]. It should be mentioned that while the spectral overlap of Tb^3+^ emission with chlorin e_6_ absorption could be poor, extremely high values of the donor (i.e., Tb^3+^) excited state lifetime could still lead to efficient EEET at significant distances.

**Fig. 8:**
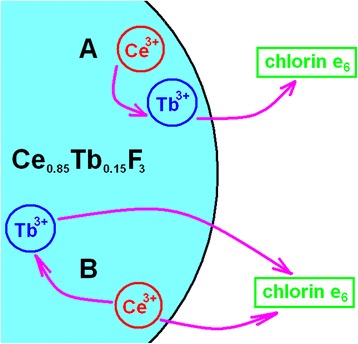
Schematic representation of the supposed EEET pathways (*arrows*) between perturbed Ce^3+^ ions, Tb^3+^ ions, and chlorin e_6_ molecules bound to Ce_0.85_Tb_0.15_F_3_ NP (CTAB shell is not presented for simplicity)

To analyze in more details the quenching of Tb^3+^ emission, let us look at the components of the three-exponential fit of the decay curve for the most intensive Tb^3+^ luminescence band at 543 nm (Table 1). It is seen that the decay times of all three components decrease upon the addition of chlorin e_6_ pointing to EEET from Tb^3+^ to chlorin e_6_ for all of them. EEET efficiency calculated according to (3) for all three components turns out to be the highest (0.42 for 2 μM of chlorin e_6_) for the shortest component *τ*_1_ and the lowest (but still as high as 0.15 for 2 μM of chlorin e_6_) for the longest component *τ*_3_. At the same time, while the amplitude *A*_1_ of the shortest component stays about the same at different concentrations of chlorin e_6_, that of the medium one *A*_2_ does not change at 2 μM of chlorin e_6_ and decreases almost twice at its highest concentration of 10 μM. At the same time, the amplitude *A*_3_ of the longest component decreases the most strongly (more than twice at 2 μM and more than 10 times at 10 μM of chlorin e_6_). We could thus suppose that the luminescence intensity corresponding to the shortest component *τ*_1_ (and the medium one *τ*_2_ at the low concentrations of chlorin e_6_) decreases mainly due to EEET from Tb^3+^ to chlorin e_6_. At the same time, the intensity of the longest component *τ*_3_ diminishes due to both (i) EEET from Tb^3+^ to chlorin e_6_ (that reduces τ_3_) and (ii) decreased population of Tb^3+^ excited states due to competition of EEET from Ce^3+^ to Tb^3+^ with that from Ce^3+^ to chlorin e_6_ (that reduces *A*_3_). We could further speculate that the short-time emitting Tb^3+^ ions receive excitations without competition with Ce^3+^-to-chlorin e_6_ EEET. At the same time, these short-time emitting Tb^3+^ ions surprisingly demonstrate the highest Tb^3+^-to-chlorin e_6_ EEET efficiency. The possible explanation could be as follows (Fig. 8). We could suppose that Ce^3+^ perturbed states (that demonstrate luminescence near 355 nm (Fig. 5)) are mostly connected with Ce^3+^ ions situated close to the surface of the NP. In this case, Tb^3+^ ions with shorter decay times are supposed to be situated near the NP surface as well and close to perturbed Ce^3+^ ions (Fig. 8, A). This results in (i) high Ce^3+^-to-Tb^3+^ EEET rate (due to short distance) leaving no place for competition by Ce^3+^-to-chlorin e_6_ EEET, and (ii) high rate of subsequent Tb^3+^-to-chlorin e_6_ EEET. At the same time, Tb^3+^ ions with longer luminescence decay times are supposed to be situated further from the NP surface (generally, higher distance from surface means lower impact of various quenchers that is consistent with higher luminescence decay times); they receive excitations from the perturbed Ce^3+^ ions which do not neighbor short-time emitting surface Tb^3+^ ions in close proximity (Fig. 8, B). This results in (i) lower Ce^3+^-to-Tb^3+^ EEET rate that permits partial redirection of Ce^3+^ excitation flow to the Ce^3+^-to-chlorin e_6_ EEET pathway and (ii) lower rate of subsequent Tb^3+^-to-chlorin e_6_ EEET.

**Table 1:** Parameters of the three-exponential fit of the decay curve of Tb^3+^ luminescence band at 543 nm for Ce_0.85_Tb_0.15_F_3_ NP in the presence of 0.05 mg/mL CTAB and Ce_0.85_Tb_0.15_F_3_ NP-CTAB-chlorin e_6_ nanosystem (2, 5, or 10 μM of chlorin e_6_; 0.05 mg/mL CTAB)

Chlorin e_6_ concentration	*τ*_1_, ms	*A*_1_, a.u.	*B*_1_	*E*_*Tb−Ce6*_ *(τ*_*1*_ *)*	*τ*_2_, ms	*A*_2_, a.u.	*B*_2_	*E*_*Tb−Ce6*_ *(τ*_*2*_ *)*	*τ*_3_, ms	*A*_3_, a.u.	*B*_3_	*E*_*Tb−Ce6*_ *(τ*_*3*_ *)*
0 μM	0.69	34	0.02	*–*	2.35	81	0.17	*–*	6.13	147	0.81	*–*
2 μM	0.40	48	0.04	0.42	1.71	84	0.29	0.27	5.18	64	0.67	0.15
5 μM	0.31	47	0.06	0.54	1.32	63	0.36	0.44	4.51	29	0.58	0.27
10 μM	0.27	44	0.10	0.61	1.06	46	0.42	0.55	3.98	14	0.48	0.35

*τ*_1_, *τ*_2_, and *τ*_3_ are the decay times; *A*_1_, *A*_2_, and *A*_3_ are the amplitudes; *B*_1_, *B*_2_, and *B*_3_ are the relative intensities (calculated as *B*_*i*_ = *A*_*i*_ × *τ*_*i*_/Σ *A*_*i*_ × *τ*_*i*_, *i* = 1, 2, 3) of the three-exponential fit; *E*_Tb−Ce6_(*τ*_*i*_) is the EEET efficiency calculated for the corresponding decay component according to the expression (3)

Basing on the above observations, photophysics processes in the Ce_0.85_Tb_0.15_F_3_ NP-CTAB-chlorin e_6_ nanosystem could be as follows. Suggesting that perturbed Ce^3+^ ions are mainly localized near to the NP surface, for the part of these ions situated close to Tb^3+^ ones EEET only to Tb^3+^ takes place. For the other Ce^3+^ ions, competition between EEET to Tb^3+^ (localized at higher distance from the NP surface) and to chlorin e_6_ exists. For both cases, excitations of Tb^3+^ are further transferred to chlorin e_6_.

## Conclusions

1. Chlorin e_6_ molecules bind to Ce_0.85_Tb_0.15_F_3_ NP in the presence of CTAB forming thus the Ce_0.85_Tb_0.15_F_3_ NP-CTAB-chlorin e_6_ nanosystem. We consider that binding occurs via chlorin e_6_ embedding in the shell formed around NP by CTAB molecules.
2. In the Ce_0.85_Tb_0.15_F_3_ NP-CTAB-chlorin e_6_ nanosystem, electronic excitation energy transfer from Ce ^3+^ to chlorin e_6_ takes place both directly (with the 0.33 efficiency for 2 μM chlorin e_6_) and via Tb^3+^.

## Abbreviations

CTAB: Cetrimonium bromide
DLS: Dynamic light scattering
EEET: Electronic excitation energy transfer
NP: Nanoparticles
PDT: Photodynamic therapy
TEM: Transmission electron microscopy
